# Integrating Biological Motion: The Role of Grouping in the Perception of Point-Light Actions

**DOI:** 10.1371/journal.pone.0025867

**Published:** 2011-10-03

**Authors:** Ervin Poljac, Karl Verfaillie, Johan Wagemans

**Affiliations:** Laboratory of Experimental Psychology, University of Leuven (K.U. Leuven), Leuven, Belgium; University of Minnesota, United States of America

## Abstract

The human visual system is highly sensitive to biological motion and manages to organize even a highly reduced point-light stimulus into a vivid percept of human action. The current study investigated to what extent the origin of this saliency of point-light displays is related to its intrinsic Gestalt qualities. In particular, we studied whether biological motion perception is facilitated when the elements can be grouped according to good continuation and similarity as Gestalt principles of perceptual organization. We found that both grouping principles enhanced biological motion perception but their effects differed when stimuli were inverted. These results provide evidence that Gestalt principles of good continuity and similarity also apply to more complex and dynamic meaningful stimuli.

## Introduction

Human vision has highly efficient mechanisms to recognize actions of others rapidly and without apparent effort, even when the stimulus is defined solely by a few moving light points [1]. Although only some of the stimulus properties are kept in this case, such as the global shape and the motion trajectories of these points, observers experience compelling motion of a human figure engaged in a specific activity. The perception of point-light stimuli provides an excellent example of the Gestalt principle that the whole is more than the sum of its parts. How exactly the visual system manages to organize this highly reduced stimulus into a vivid percept of human action and what mechanisms are responsible for the perception of biological motion still puzzles vision scientists.

Recent studies that have attempted to identify key features of the point-light stimulus largely focused on the importance of local motion signals for the perception of biological motion. For instance, Casile and Giese [2] argued that the integration of individual point-light elements into a percept of a walker might be accomplished by detecting mid-level motion features, while precise position information from the impoverished stimuli is not necessary (spatial localization is relatively coarse). Relative motion of the elements that make up the stimulus is considered to be crucial and in particular the opponent motion of the pairs of ankles and wrists. Troje and Westhof [3] narrowed down the crucial features even further to the motion of the feet, which is essential in differentiating biological from non-biological motion [also 4], [5].

Beside the rather strong evidence that motion is important, the relative contribution of shape information has also been emphasized. Several studies found evidence for the accurate processing of biological motion stimuli in which local motion was removed by jittering the individual dots' locations from frame to frame along the limbs [6], [7]. Studies that used static point-light displays (snapshots) have also shown that the detection of walkers is possible without any motion cues [8] and that performance can be further facilitated by additional form cues [9].

While the controversy about key features and their relative contribution to biological motion perception still remains unresolved, the apparent contradiction might at least partly be reconciled: Inconsistent findings often originate in the nature of the tasks that are used and the availability of cues that are required for optimal performance in a particular task [10]. Local motions may be efficiently utilized to estimate the direction of point-light walkers in the lateral view for instance; yet to recognize the action represented by such a figure [11], [12], the judgment probably depends on a more global pattern. In the latter case, the local elements probably need to be incorporated into a configural representation of the figure as a whole to enable its recognition. This global structure can be constructed from the relative motion signals of the constituent dots that convey global information [13]. In static presentations, however, the point-light elements are successfully integrated into a representation of a human figure, clearly without any motion [8], [10]. This indicates a possible mechanism that involves cues other than motion signals that allow the integration of the elements into a coherent human figure.

For both, representations based on dynamic and representations based on static information, the visual system manages to group the elements into a meaningful whole: How are the disparate dots making up a point-light stimulus connected? This recovery of the connectivity structure of a point-light action is a form of grouping. Grouping and perceptual organization were central on the research agenda of Gestalt psychology. Indeed, Gestalt psychologists described a number of laws in visual perception, defining what is necessary to group a number of elements into one object. A stimulus object that can be organized according to these rules represents a ‘good Gestalt’ and hence the object will be salient and easily recognized. The question arises to what extent well documented Gestalt laws of perceptual grouping facilitate the recovery of the connectivity structure during the perception of biological motion. To investigate this issue, we implemented two well-known Gestalt principles, that of good continuation and similarity, by presenting a human walker by means of oriented Gabor patches instead of non-oriented dots.

Wertheimer [14] was probably the first to describe that spatially aligned neighbouring features induce a percept of continuous contour. The collinearity of the elements reveals the underlying shape of the object and facilitates its identification [15]–[19] and it is most commonly interpreted as a consequence of neuronal interactions in low-level visual areas. Lateral connections between V1 neurons might provide the neural circuitry for the principle of good continuation [20], as connections between neurons tuned to the same orientation are more numerous [21], in particular those with spatially aligned receptive fields [22].

Collinearity is probably the strongest grouping cue in the context of biological motion perception, since the local orientations of the elements are consistent with the underlying shape to which the elements belong. The alignment of the elements might thus reinforce the shape signals and enhance the perception of a human figure.

In addition, similar elements also tend to group together, regardless of the origin of their resemblance, the motion pattern, luminance, or orientation in space. In our study, we introduced similarity into the point-light stimulus by orienting the Gabor patches making up the walker in the same direction (similarity by isolinearity). This similarity could support the segmentation of elements from the display and hence facilitate their interpretation. Isolinearity is most likely a less strong grouping principle in this context than collinearity. Although all elements have the same orientation, their orientation does not reveal the underlying structure at a local level, and therefore we might expect lower rates of recognition. By employing these grouping cues, we can investigate how much they might facilitate the recovery of the connectivity structure of a point-light figure.

An additional aim of the present study is to further investigate the configural nature of biological motion processing and the possible role of grouping cues in configural processing. Tadin, Lappin, Blake, & Grossman [23] showed that perceiving the elements of a point-light figure as organized in a global form is beneficial for the representation of elements' relative positions and motions. Such an advantage is observable in upright figures, but not in inverted ones. Examining the effect of inversion is a well established means to study configural perception. Indeed, the spatiotemporal structure remains the same when a point-light walker is inverted, but the underlying shape of a human figure is much more difficult to perceive, as if it loses the emergent properties, making the grouping of the elements certainly not as immediate and compelling [24]–[27]. The grouping cues might boost form perception in both dynamic and static presentations of the upright point-light walkers, reflecting their involvement in the processing of biological motion, but they may have different effects on inverted figures. Similar to the role it has in face processing [28]–[30], inversion is a way to investigate the global, configural character of biological motion perception.

## Methods

### Participants

Fifteen participants (9 female) from the undergraduate psychology program at the University of Leuven conducted the experiment for course credit. They all had normal or corrected-to-normal vision. The study was conducted in accordance with the ethical standards laid down in the 1964 Declaration of Helsinki and was approved by the Ethics Committee of the Faculty of Psychology, University of Leuven. All participants gave their written informed consent.

### Stimuli

**Figure 1:**
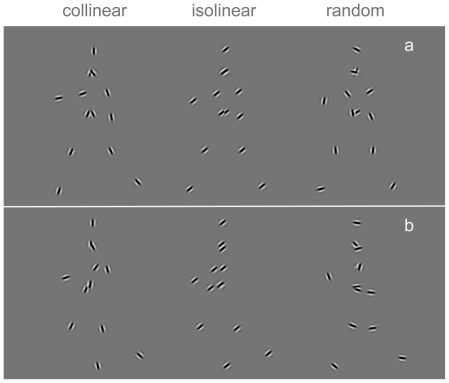
Walking figures represented by Gabor patches. The figures that represented human walkers differed with regard to the orientations of the constituent Gabor elements: The orientation either corresponded to the body-lines (collinear), all Gabor elements had the same spatial orientation (isolinear), or the orientation was randomly chosen in the first frame (random) for each element (a). In the temporally scrambled counterparts (b) of the walkers, the initial frame of each separate Gabor element was randomly chosen within a walking cycle and had the same motion as in the corresponding elements of the intact figure.

The experimental stimuli were generated using Matlab (Mathworks Inc.) and Psychophysics toolbox extensions [31], [32]. Point-light human motion sequences were adopted from Vanrie and Verfaillie [12], [33], which are based on motion capture data (Qualisys MacReflex). Each gait cycle duration was 1 s and contained 60 frames (an interpolation procedure was employed to calculate the positions of each individual Gabor patch to the used frame rate). The points constituting the walking figure were replaced by small Gabor patches, each with a spatial frequency of 3 cycles/deg (and a standard deviation of the Gaussian envelope of 0.1°). Three different walkers with regard to the orientation of Gabor patches were created (Figure 1a, Movie S1, Movie S2, Movie S3). In the *collinear* condition, the orientation of each Gabor patch was calculated relative to the underlying body-parts (as in a stick-figure presentation of the human figure), aligned with that body-part and was thus updated each frame. The orientation change was the largest for the Gabor patches representing wrists and feet, and smaller for some other patches, such as shoulders and hips. In the *isolinear* condition, all Gabor patches that constitute the walker had the same spatial orientation. In the *random* condition, the orientation of each Gabor patch was randomly chosen in the first frame and was updated each frame such that the change in orientation of individual Gabors across the whole presentation was the same as in corresponding Gabors in collinear walkers. The orientation of the head patch in the collinear condition was fixed to the vertical orientation. A scrambled counterpart was created for each of the three walker conditions, in which the initial frame of each separate Gabor element was randomly chosen within a walking cycle, but the motion remained the same as in the corresponding elements of the intact figure, as did the Gabor orientations (Figure 1b). For all upright walkers, we also created an inverted version by rotating the whole image by 180°. If two Gabor patches overlapped during the presentation, the average luminance value was taken, so that their ‘distance in depth’ could not be distinguished.

### Procedure

Participants were seated approximately 57 cm from the screen (Dell 19″ CRT monitor) on which stimuli were displayed at a resolution of 640×480 at 60 Hz, resulting in the stimulus subtending about 5° of visual angle in height and 2° in width. Background was light-gray with a luminance of 60 cd/m^2^. The basic design employed a two-alternative forced choice paradigm with a temporal succession of two presentations, one containing an intact point-light walker (Figure 1a) and the other a temporally scrambled version (Figure 1b) with the same number of elements. Subjects had to indicate in which of the two successive presentations of each trial the intact representation of a human figure appeared, by pressing a key.

Unlike the standard detection in noise task [34]–[36], we varied the number of elements that make up the walker on a trial-by-trial basis, from one to seven, out of thirteen possible locations at which Gabor patches could be positioned (head, shoulders, elbows, wrists, hips, knees, and ankles). The reason we avoided the use of noise is the possible difficulty related to the orientation of the noise elements. Because the three conditions differed with regard to the orientation of the Gabor elements, it was not possible to have one type of noise condition, and choosing different orientations for the noise elements in the three conditions would clearly complicate the comparisons. Each Gabor patch had a limited-lifetime presentation, after which it was replaced by another patch at a randomly chosen location of the thirteen possible locations. The figures were always presented as walking on a treadmill, with the walking direction (to the left or to the right) randomized across trials. The initial point-light frame was randomized for each trial and continued one full walking cycle. In this way we tried to avoid possible observers' predictions and balanced for potential differences that might originate in the degree of task difficulty known to be related to the phase of the gait cycle [9]. The stimulus position on the screen was jittered randomly in each trial by up to 3° relative to the centre, to prevent that local motion cues of individual elements could be used to solve the task. The six different versions of the walker (three different orientations of Gabor patches and their inverted versions) were presented in blocks, with breaks between the blocks and the blocks balanced across subjects to control for order effects. Practice trials were included to verify that participants could perceive the figure represented by Gabor elements at maximal number of elements. Each condition was presented 40 times, for a total of 240 trials. Participants were given feedback about their performance after each trial. The method of constant stimuli was used to estimate 75% performance thresholds.

### Experiment 1. Enhancing the grouping cues in dynamic presentations

To examine whether a particular arrangement (orientation) of the Gabor patches affects the perception of biological motion, we first employed dynamic presentations of the experimental stimuli, with collinear, isolinear, and random element arrangements within a walker, as well as their inverted counterparts. Participants had to indicate which of the two presentations in each trial contained an intact representation of a human figure by means of a key-press. The collinear orientation of the Gabor patches might facilitate grouping and enhance the perception of the underlying body connectivity structure. The isolinear stimulus does not give clues to the underlying shape, at least not at the local level, but does contain other cues that can support the integration of the individual elements. The similarity of the Gabor patches with regard to their orientation might be employed by the visual system to integrate them into an organized global shape and hence facilitate perception.

### Experiment 2. Static presentations

To further investigate the contribution of grouping in biological motion perception, we employed static presentations (i.e., snapshots) of postures from the walking cycle [8], [10]. In this case the judgment relies solely upon the form cues. Nine participants (five female) of the initial group of fifteen students completed a paradigm similar in all respects to that of Experiment 1, except for the snapshot presentations of a walker with the duration of 1 s, instead of dynamic presentations. If the integration cues introduced through the manipulation the elements' orientations are important for the creation of the percept of biological motion, and if they indeed convey information about the form, we should be able to observe differences for our three walkers, as well as for their inverted versions.

### Experiment 3. Different viewpoint

Usually, biological motion has the status of a special stimulus for the visual system. However, paradigms used to investigate its perception most often involved a point-light representation of a human figure in one particular orientation (lateral presentation). Therefore, the question of generalization and ‘ecological validity’ issues were often raised [9], as the effects observed in studies employing a specific viewpoint do not have to hold for other viewpoints [37]. To enable the generalization of the effects at least to a certain extent to other representations of biological motion, we performed an experiment similar to Experiment 1, with the Gabor-patch representations of the human figures as seen from ¾ viewpoint (45°), other things being the same.

### Experiment 4. Baseline: the standard point-light display

To further explore the contribution of the two implemented organizational principles, we asked the participants to repeat the same experiment with 45° view, while the figures were defined by small bright dots, as in the classical representation of the point-light walker. Their size was approximately 0.1° visual angle. The point-light stimulus does not contain (local) cues as the collinear and isolinear condition and could be thus used as a base-line. This was necessary to examine whether the manipulations in previous conditions lead to improvement or decrease in performance relative to this condition.

## Results

Participants had to indicate in which of the two presentations of each trial they perceived an intact point-light representation of a human figure, while we manipulated the orientation of individual Gabor-elements that constituted the figure. For data analysis, 75% correct performance thresholds were estimated by fitting a Gaussian distribution to the data (maximum likelihood method, [38]).

### Experiment 1. Enhancing the grouping cues in dynamic presentations

**Figure 2:**
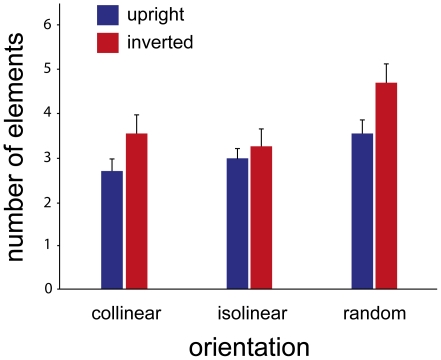
Performance for the six dynamic conditions. The blue and red bars represent the average number of elements required for 75% correct judgment for the upright and inverted versions of the walkers, respectively, for the three orientation arrangements: collinear, isolinear and random. Collinear and isolinear arrangements result in a better performance than random orientation of the Gabor patches. There is an overall effect of inversion, which is stronger in the collinear and random arrangements than in the isolinear one. The error bars denote 95% confidence intervals.

Three different arrangements with respect to the orientation of the Gabor elements that make up the point-light stimulus were presented, as well as their inverted versions. The participants were asked to judge which presentation contained an intact walking figure, while the number of elements varied on a trial-by-trial basis. Figure 2 shows the performance for the six experimental conditions, depicting the average number of elements required to discriminate the intact walker from its phase-scrambled version at 75% correct performance level. Statistical analysis (repeated measures ANOVA) revealed a significant effect of the orientation of the Gabor patches (F(2,13) = 24.25; p<0.001). While the collinear and isolinear orientations did not differ significantly from each other (F(1,14) = 3.37; p = 0.09), the random arrangement was significantly more difficult; the participants needed more elements to make the correct judgment compared to the other two orientations (relative to collinear F(1,14) = 20.56; p<0.001, relative to isolinear F(1,14) = 6.36; p = 0.02). There was also a considerable effect of inversion across the conditions (F(1,14) = 99.55; p<0.01). However, we observed a trend towards an interaction effect (p = 0.06): The inversion effect (i.e., the difference between the upright and the inverted condition) was much smaller for the isolinear condition (F(1,14) = 3.46; p = 0.08) than for the collinear and random conditions (F(1,14) = 53.90; p<0.001 and F(1,14) = 59.08; p<0.001, respectively). Moreover, this interaction was clear if the inversion effect was analysed separately for the isolinear and collinear conditions (F(1,14) = 13.03; p<0.001), or for the isolinear and random conditions (F(1,14) = 14.96; p<0.001). There was no difference between the inversion effects when the collinear and random conditions were compared (F(1,14) = 2.82; p = 0.12).

### Experiment 2. Static presentations

**Figure 3:**
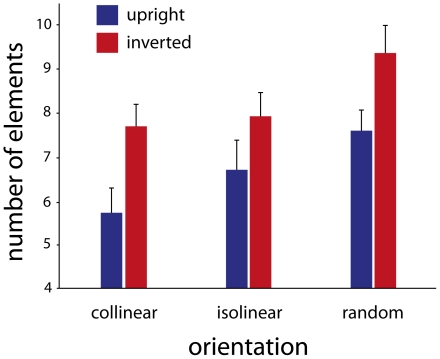
Performance for the static presentations. The bars represent the average number of elements required to make a 75% correct judgment. For the random arrangement of the elements participants needed significantly more elements than for the collinear and isolinear arrangements, in both upright and inverted conditions. The inversion effect was pronounced and considerably stronger than for the dynamic presentations, and unlike the dynamic conditions, it was also observed in the isolinear condition. The error bars denote 95% confidence intervals.

The integration of point-lights into the representation of a walker is often assumed to require both spatial and temporal information. We asked the participants to discriminate intact and scrambled presentations of experimental stimuli, this time displaying only static snapshots. On the one hand, the average number of elements required to make a correct judgment increased strongly compared to the dynamic presentations (from about 3.5 to about 7 elements, on average; see Figure 3), indicating that spatiotemporal information indeed is important for the perception of biological motion. On the other hand, the data show a comparable pattern to that in Experiment 1. Here too, for the random arrangement of the elements participants needed significantly more elements than for the other two conditions (F(2,7) = 42.25; p<0.01), in upright and inverted conditions. There was again an inversion effect, but it was considerably stronger than in Experiment 1 (F(1,26) = 145.73; p<0.001). Both collinear and isolinear stimuli showed stronger grouping than random orientations of the Gabor elements. The grouping effect in the upright condition was stronger for the collinear than for the isolinear stimuli (F(1,8) = 5.53; p = 0.03), but this difference disappeared with inverted stimuli (F(1,8) = 1.29; p = 0.28). Nevertheless, the interaction effect on the whole was not significant, although there was a trend when isolinear and collinear conditions were compared for the inversion effects (F(1,8) = 4.35; p = 0.08).

### Experiment 3. Different viewpoint

**Figure 4:**
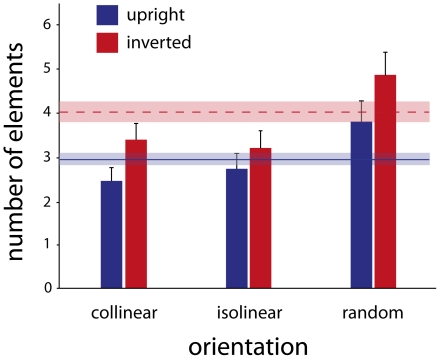
Results for the 3/4 viewpoint and classic point-light display. The average subjects' performance for the ¾ viewpoint was very similar to Experiment 1 with regard to the average number of elements required for the discrimination task, as well as the same interesting absence of inversion effect in the isolinear condition. The error bars denote 95% confidence intervals. The solid blue and dashed red lines denote the subjects' performance for the upright and inverted walkers, respectively, with regard to the ‘classic’ point-light display. The performance is worse than for the collinear Gabor-walker, but better than the random condition.

To examine whether the findings generalize to other views than the sagittal view, participants performed the same task as in the previous two experiments, but the stimulus now was rotated along its vertical axis, as if it was looked at under a viewing angle of 45°. Figure 4 summarizes the data of the same nine participants as in previous experiment. Statistical analysis showed results very similar to Experiment 1. The orientation of the walker's Gabor patches had an effect on the number of elements that the participants needed to make a correct judgment (F(2,7) = 35.24; p<.01). Again, participants needed less Gabor elements in the collinear and the isolinear condition than in the random condition. The interaction effect between the orientation and inversion effect was significant now (F(2,7) = 5.69; p = .03), which in particular applied to isolinear vs. collinear arrangements (F(1,8) = 11.58; p<.01): The inversion effect was less pronounced in the isolinear condition than in the collinear condition.

### Experiment 4. Baseline: the standard point-light display

Participants repeated the experiment, while the figures were made up of dots, as in the classical representation of the point-light walker. As depicted in Figure 4, the perception of both upright and inverted point-light walkers outperformed the random condition (F(1,8) = 20.7; p<.01 and F(1,8) = 35.39; p<0.001, respectively). There was no significant difference between the isolinear and the classical walkers in the upright condition (F(1,8) = 1.45; p = .17), but their inverted versions did differ (F(1,8) = 18.9; p<.01). The performance was also better for the collinear walker, upright and inverted, compared to the classical point-light display (F(1,8) = 14.9; p<.01 and F(1,8) = 6.12; p = .04).

## Discussion

The role of grouping in visual perception has been repeatedly demonstrated, but not until very recently has its importance been shown for the perception and recognition of meaningful objects [17], [18]. The current study investigated whether grouping is important for the perception of biological motion stimuli, more specifically for the integration of elements that constitute a point-light walker. The results provide evidence that the recovery of the connectivity structure of a point-light figure is facilitated when the stimulus elements can be grouped according to Gestalt laws of perceptual organization (good continuation and similarity).

We found that a collinear arrangement of the Gabor elements facilitated biological motion perception, as reflected in a better performance than when the elements were randomly oriented. One likely interpretation for this finding is that the random local orientation of the elements is less informative of the underlying shape of the figure, while the collinear orientations of the Gabor patches help to indicate the local underlying form of the figure. This is in line with previous findings that contour integration is strongest when Gabor elements are aligned with the underlying contour [39]. Our finding provides support for the notion that the principle of good continuation can also facilitate biological motion perception. Thirkettle et al. [10] manipulated the strength of opponent motion signals by orienting the Gabor patches that defined the human figure orthogonally to their opponent motion paths, which resulted in enhanced perception. However, this manipulation coincided with the alignment of the neighbouring Gabor patches along the limbs, which is in accordance with the good continuation principle. The authors concluded that there is a vital role of form information in processing point-light displays, but similar to our stimuli, their manipulation was probably beneficial for contour integration mechanisms. Increased congruency between the elements and the underlying shape resulted in enhanced perception. In addition, our study supports previous findings that an offset or misalignment of the Gabor elements relative to the contour of the object makes its identification more difficult [18]. Pelli et al. [17] demonstrated the role of grouping in identification of meaningful objects, explaining the effects by the increase of stimulus complexity when there is an offset, while grouping reduces it and consequently improves the efficiency of the recognition (for an alternative view on the interplay between stimulus complexity, perceptual grouping, and object recognition, see [40], [41]).

The same collinear arrangement of our Gabor-walkers was also better when compared to the classic point-light walker, although this comparison should be made with caution, taking into account that local motion signals are different for isotropic dots and oriented Gabor patches, as well as their contrasts, despite the similar motion paths. A direct and fully parametric comparison was not the goal of this study, we just added the standard condition as a benchmark to be able to relate it to the more traditional stimuli and paradigms in the literature.

Similar to the random orientations, when all Gabor patches are spatially uniformly oriented, as in our isolinear condition, they do not immediately reveal the structure of the figure. Still, compared to the random orientations we observed a facilitatory effect of this arrangement for upright stimuli, with performance at approximately the same level as for the collinearly aligned elements. In addition, the performance is slightly (but not significantly) better than for the classic point-light walker. The Gestalt principle of similarity states that elements with similar features tend to be grouped together [14], [42], [43] and it is evidently a valid principle in biological motion perception too. Hunt and Halper [44] showed that if the points of light that define a walker were replaced by different elements, their integration into a percept of a human figure became more difficult. The authors replaced the dots by a range of different objects, some similar to each other, others more dissimilar. For the similar elements (e.g., all letters ‘A’) the perception of the figure was only slightly compromised, but when the elements were replaced by all different unique objects (e.g., colour pictures of everyday objects), the percept was almost entirely disintegrated. Although the authors did not systematically manipulate the implementation of different Gestalt principles or examined their role for the perception, this finding is consistent with the current study, demonstrating an increased performance resulting from grouping based on the similarity of the elements, compared to conditions with unique, dissimilar objects.

In the present study, all dynamic displays contained the same motion irrespective of the local orientation of the individual elements. Motion is a useful cue in the perception of point-light displays, especially the opponent motion of the pairs of limbs, which is often put forward as a key feature for the recognition of biological motion as such (e.g., [2]). One interesting characteristic of the point-light displays related to the opponent motion is the simultaneous change in the direction of motion of the opponent pairs of elements. This in itself might also represent a valid grouping cue. Lee and Blake [45] showed that synchronized change of orientation of a number of elements is a strong cue for grouping those elements and interpreting them as belonging to the same object.

To disentangle the influence of orientation cues from motion, we also employed static displays. In his 1973 report Johansson suggested that static point-light displays (‘snapshots’) do not contain enough information to correctly interpret an action, and research that followed largely assumed that motion is indeed crucial. However, Thirkettle et al. [10] found that at least for walking it is possible to identify figures from static displays, as well as that the orientation of Gabor elements affects perception, as a collinear arrangement (orthogonal to the motion vectors) resulted in more accurate perception. Approaches other than psychophysics also provided support for this conclusion. Lange, Georg, & Lappe [46], for example, used a template-matching method to demonstrate that it is possible to derive biological motion from static point light displays, while neuroimaging studies have identified biological motion areas responsive to static postures [47], [48]. Our study confirms that the perception from static displays is possible and that it is enhanced by grouping cues. We found a clear advantage of collinearly and isolinearly oriented Gabors compared to randomly oriented patches. In addition, it is important to emphasize that elements in our dynamic presentations had a very short limited lifetime after which they were replaced by different elements positioned at other positions on the figure. Hence, elements were presented at several locations throughout a trial, which gave the impression of more elements being perceptually present than their actual number in each individual frame. This consideration means that a comparison of the number of elements between the static and dynamic condition should be done carefully, especially when this is used as a measure of performance.

A characteristic inversion effect was revealed in worse performance in both dynamic and static displays in our study. Once the walker is presented upside-down, the human figure is not as easily recognized as such anymore [25], [26], despite the fact that all the relative motions of the element dots are preserved. When inverted, the stimulus seems to lose the emergent properties and observers perceive the motion of individual elements without a clear global structure [24]–[26]. The effects of inversion observed in our study were comparable to previous reports; however, one unanticipated result is the observed level of performance for the isolinear dynamic condition. While the collinear arrangement showed the usual decrease in performance as a result of stimulus inversion, we did not observe such a strong decrement in the isolinear condition. This differential effect on the perception might indicate a dissimilar processing mechanism for the two arrangements: Perceptual grouping seems to be more of a configurational nature in the collinear condition than in the isolinear condition.

The primary goal of Gestalt psychologists was to understand and define principles responsible for the perceptual organization of our visual impressions of everyday objects (e.g., Koffka's famous question: “Why do things look the way they do?”). Stimuli used in most studies of perceptual grouping were usually artificial patterns, shapes or contours and in that sense they represented meaningless objects. While their use in the study of grouping is valuable, the principles should also apply to more complex, meaningful stimuli. We here show that the perception of biological motion, represented by point-light figures, is also enhanced when perceptual grouping of the elements is enhanced.

## Supporting Information

Movie S1 **The collinear walker.** The orientation of Gabor elements are corresponds to the body-lines.
(WMV)

Movie S2 **The isolinear walker.** All Gabor elements have the same spatial orientation.
(WMV)

Movie S3 **The random walker.** The orientation randomly chosen in the first frame for each element.
(WMV)
